# Natural and anthropogenic drivers of cub recruitment in a large carnivore

**DOI:** 10.1002/ece3.4180

**Published:** 2018-06-17

**Authors:** Femke Broekhuis

**Affiliations:** ^1^ Kenya Wildlife Trust Nairobi Kenya; ^2^ Wildlife Conservation Research Unit Department of Zoology University of Oxford Recanati‐Kaplan Centre Tubney UK

**Keywords:** cheetah, cub recruitment, habitat, lion, survival, tourism

## Abstract

Recruitment is a critical parameter governing population dynamics and influences population persistence. Understanding the drivers of recruitment is therefore important for conservation, especially for long‐lived mammals such as large carnivores, which have low reproductive rates, rendering them prone to extinction. Using cheetahs (*Acinonyx jubatus*) as a model species, I investigated the variation in cub recruitment in relation to habitat and the abundance of tourists and predators. Per litter, female cheetahs on average raised 1.71 ± 1.35 cubs to independence, but this varied depending on the presence of open habitat and the abundance of tourists, both of which had a negative effect on cub recruitment. More specifically, female cheetahs that were mostly found in open habitats on average raised 1.69 ± 0.14 cubs per litter to independence compared to 3.04 ± 0.26 cubs in denser habitat. Similarly, female cheetahs that were exposed to high tourist abundance on average raised 0.21 ± 0.72 cubs to independence compared to 2.32 ± 0.11 cubs in low tourism areas. Neither lion nor spotted hyaena abundance had an impact on the number of cubs that were recruited. Based on these findings, I recommend that the importance of a heterogeneous environment should be taken into consideration in habitat management, restoration efforts, and reintroduction programs. In addition, tourist quotas should be put in place in high visitation areas and strict wildlife viewing guidelines, such as number of vehicles, tourist behavior, time spent, and distance to a sighting, should be enforced. Cub recruitment is an important component of species persistence and incorporating these findings could aid conservation efforts for species that are increasingly under threat.

## 1 INTRODUCTION

Recruitment, defined as offspring survival to independence, is a key parameter governing population dynamics, such as growth rate, which is vital for population persistence (Ozgul, Armitage, Blumstein, & Oli, 2006). Offspring recruitment can be influenced by a multitude of factors (Coulson, Albon, Pilkington, & Clutton‐Brock, 1999) such as disease (Mech & Goyal, 1995), food availability (Höner, Wachter, East, Runyoro, & Hofer, 2005), habitat (Mosser, Fryxell, Eberly, & Packer, 2009; Waser, Elliott, Creel, & Creel, 1995), and intra‐ and interspecific competition and predation (Creel & Creel, 1998; Watts & Holekamp, 2009). Anthropogenic activity can also affect recruitment. For example, harvesting decreased recruitment in wolves (*Canis lupus*) in Alaska (Ausband, Stansbury, Stenglein, Struthers, & Waits, 2015) and increased human access to protected areas had a negative effect on cub survival of tigers (*Panthera tigris*) in Russia (Kerley et al., 2002). Similarly, climate change can cause declines in offspring recruitment through changes in habitat (Rode, Amstrup, & Regehr, 2010) and behavior (Woodroffe, Groom, & McNutt, 2017). Therefore, understanding which factors influence offspring recruitment is important for conservation. This is especially the case for long‐lived mammals, such as large carnivores (Gaillard, Festa‐Bianchet, & Yoccoz, 1998; Heppell, Caswell, & Crowder, 2000), as they generally occur at low densities and have low reproductive rates which renders them prone to extinction (Purvis, Gittleman, Cowlishaw, & Mace, 2000).

Cheetahs (*Acinonyx jubatus*) have experienced drastic population declines and with only ~7,000 mature individuals left in Africa (Durant et al., 2017), understanding what influences population dynamics is crucial. Recruitment can positively influence the population growth of cheetahs (Johnson et al., 2013), but while litter sizes for cheetahs generally range between one to six cubs, their juvenile mortality is high, and as a result, their cub recruitment is low (Laurenson, 1994). In the Serengeti National Park in Tanzania, less than 5% of cubs reach independence (Laurenson, 1994), while in the Kgalagadi Transfrontier Park in South Africa/Botswana, this is estimated to be 28.9% (Mills & Mills, 2017). Cheetah cubs can succumb to various factors including abandonment, poor health, and fires. However, it is predation by other predators, especially lions (*P. leo*) and spotted hyaenas (*Crocuta crocuta*), that is the predominant cause of cub mortality across sites (Laurenson, 1994; Mills & Mills, 2017) and lion abundance has been shown to be negatively correlated to the recruitment of cheetah cubs (Durant, Kelly, & Caro, 2004). However, the occurrence of predator‐induced mortality could be influenced by factors, such as vegetative cover, that provide concealment and minimizes detection by other predators (Broekhuis, Cozzi, Valeix, McNutt, & Macdonald, 2013; Rostro‐García, Kamler, & Hunter, 2015). Indeed, it has been hypothesized that vegetative cover can influence cheetah cub survival (Durant, 1998; Mills & Mills, 2017), but this has not been formally tested.

Cheetahs are considered to be a “protection reliant” species and protected areas are key to their survival especially where population growth rates outside protected areas are suppressed (Durant et al., 2017). While protected areas are generally designed to be safe havens, unchecked human pressures can have a negative impact on wildlife. Indeed, recent studies have shown that logging and livestock grazing can limit the presence of wildlife (Soofi et al., 2018), and even well‐intentioned activities, such as tourism, can have negative effects (Tablado & D’Amico, 2017). Burney (1980), for example, found that high numbers of tourists had a negative impact on cheetah hunting behavior. It is, however, unclear whether the presence of large numbers of tourists has an effect at the population level.

Using demographic data from individually recognized cheetahs in the Maasai Mara, Kenya, I test whether cheetah cub recruitment is influenced by habitat and the abundance of lions, spotted hyaenas and tourist vehicles. The Maasai Mara in Kenya is an ideal place to conduct this study as the habitat is heterogeneous, ranging from wide open grasslands to Vachellia woodland and riverine forests (Oindo, Skidmore, & De Salvo, 2003). The Maasai Mara also has high densities of cheetahs (Broekhuis & Gopalaswamy, 2016) and other predators, including lions (Elliot & Gopalaswamy, 2017) and spotted hyaenas (Green, Johnson‐Ulrich, Couraud, & Holekamp, 2018), which vary spatially. Partly because of these high densities of predators, the Maasai Mara is a popular tourist destination.

In this study, I test the hypotheses that cheetah cub recruitment varies according to differing levels of open habitat, predator abundance, and tourist abundance. More specifically, I predict that:

1. Open habitat will negatively influence the number of cubs that are recruited as open habitat provides little concealment from predators.
2. High lion and spotted hyaena abundance will negatively influence the number of cubs that are recruited as these predators are the main cause of cub mortality.
3. High tourist abundance, despite negatively influencing cheetah behavior, will have no significant influence on the number of cubs that are recruited.

## 2 METHODS

### 2.1 Study area

The study was conducted in the Maasai Mara landscape in Southwest Kenya (centered at 1°S, 35°E; elevation c. 1700 m). Data were collected in an area of approximately 2,600 km^2^ which included the Maasai Mara National Reserve and the following privately managed conservancies: Mara Triangle, Mara North, Oloisukut, Ol Chorro, Lemek, Enonkishu, Olare‐Motorogi, Naboisho, Ol Kinyei, Olarro South and Olarro North. The habitat in the study area varies greatly, ranging from open grasslands and shrubland, to riverine forests. The open grassland plains, which are dominated by *Themeda triandra*, are mostly found toward the South and West of the study area, while the North and Northeast consists mostly of Croton thickets (*Croton dichogamous*) and Vachellia woodlands (*Vachellia drepanolobium* and *V. gerrardii*). Riverine woodland can be found along the major rivers and their tributaries (Oindo et al., 2003). The Maasai Mara has a relatively high density of cheetahs with around 1.28–1.34 adults per 100 km^2^ (Broekhuis & Gopalaswamy, 2016) which is why, along with the Serengeti National Park, it is an important stronghold for the global cheetah population (Durant et al., 2017). Other predators that occur at high densities in the Maasai Mara are lions, with a density of 17 individuals (>1 year) per 100 km^2^ (Elliot & Gopalaswamy, 2017), and spotted hyaenas, with clan sizes in the Maasai Mara National Reserve ranging from 22 to 126 individuals (Green et al., 2018).

**Figure 1:**
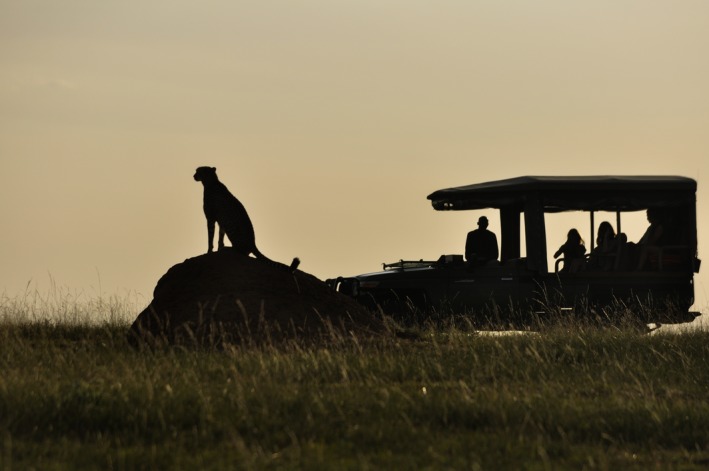
Tourists at a cheetah sighting in the Maasai Mara, Kenya. Photograph credit: Femke Broekhuis

The Maasai Mara is a popular tourist destination, and during the high season, approximately 2,700 people visit the Maasai Mara National Reserve daily, an average density of 2.7 visitors/km^2^ (Narok County Council & Trans‐Mara County Council 2012). The Maasai Mara National Reserve and the adjacent wildlife conservancies differ in their policies on tourists; the Maasai Mara National Reserve does not limit the number of tourists that enter the park per day, and there are no restrictions on the number of tourist vehicles that are allowed to be present at a predator sighting. In the Maasai Mara National Reserve it is not uncommon to see more than 30 tourist vehicles at a cheetah sighting at the same time (F. Broekhuis, unpublished data). However, in the wildlife conservancies, tourist numbers are restricted to the number of beds per conservancy and a maximum of five vehicles are generally allowed at a sighting at any given time (Figure 1).

### 2.2 Data collection

Between 1 June 2013 and 31 October 2017, a trained research team located cheetahs on an ad hoc basis using the search‐encounter method described by Broekhuis and Gopalaswamy (2016). Each female cheetah that was sighted was identified based on its unique spot pattern (Caro & Durant, 1991), and at each sighting, the date, time, and GPS location were recorded in addition to the number of dependent cubs that were present. Once cubs reached independence, the number of cubs that were recruited per litter was recorded. If a separation event was not directly observed, then the number of cubs at the last sighting was used. Only litters older than 12 months at the last sighting were included as the chance of survival between 12 months and independence is relatively high (Laurenson, 1994). To exclude the possibility that a female’s behavior might be different when she does not have cubs, only locational data when a female had cubs were used for the analysis. Only females for which more than three data points were available were included in the analysis.

For each georeferenced sighting of a female with cubs, the following data on habitat and the abundance of lions, spotted hyaenas, and tourists were extracted using ArcMap 10.3 (Environmental Systems Research Institute Inc. 2014):

*Habitat*—I created a habitat map for the study area using 30 × 30 m LandSat 8 imagery (see Table S1 for details). This habitat layer was resampled at various resolutions as there is increasing evidence that biological, ecological, and geographical processes occur at different spatial scales (Cushman & Huettmann, 2010). Because resources within home‐ranges are generally selected at a fine scale (Boyce, 2006), the proportion of open and semi‐closed habitat was calculated at six spatial scales using a moving‐window analysis in FRAGSTATS (McGarigal, Cushman, & Ene, 2012). The scales at which the habitat was resampled were 90, 180, 360, 720, 1,440, and 2,880 m which were dictated by the 30‐m resolution of the original habitat data.

*Lion, spotted hyaena and tourist abundance*—Abundance layers were created separately for lions, spotted hyaenas, and tourists using data that were collected between 1 June 2014 and 31 October 2017. While conducting search‐encounter fieldwork (Broekhuis & Gopalaswamy, 2016), the GPS locations of all lions, spotted hyaenas, and tourist vehicles were recorded, in addition to data collection effort (tracks), using an application built in Cybertracker 3 (Liebenberg, 2003). To minimize bias associated with uneven allocation of data collection effort, the following equation was used to calculate the abundance of lions, spotted hyaenas, and tourist vehicles per 2 × 2 km grid cell:

$$\text{Abundance index}=1000\times \frac{\sum z}{\sum xy}$$

where *z* is the number of individuals or vehicles, *x* the effort (distance covered in each grid cell), and *y* the detection distance which was set to 100 m. The factor of 1,000 was included to avoid very small decimal values.

### 2.3 Data analysis

The relationship between the number of cubs that were recruited per litter and habitat, lion abundance, spotted hyaena abundance, and tourist abundance were analyzed using a general linear mixed model where the mother’s ID was included as a random factor. Prior to analysis, the data were tested for normality using the Shapiro–Wilk test using the statistical software R 3.4.1 (R Development Core Team, 2016). The subsequent analysis was conducted using a two‐step approach. First, for habitat, which was calculated at different scales, a univariate scaling analysis was performed to determine which habitat category and scale had the strongest relationship with recruitment. Model selection was used to identify the most supported scale based on Akaike information criterion corrected for small sample size (AICc). The scale with the lowest AICc value was inferred to be the one which most strongly influenced recruitment and thus was retained for the next step (Table S2). Second, the effect of habitat (using the best scale from the previous step), lion, spotted hyaena, and tourist abundance on cheetah cub recruitment was determined using a multivariate analysis. The variables were tested for collinearity but as all values were |*r*| < 0.7 none of the variables were omitted from the candidate models (Dormann et al., 2013). The *a‐priori* candidate models were ranked using AICc, and relative support was assessed using Akaike weights (*wi*). When one model was superior (*w*_*i*_ **> 0.9), this was used; otherwise, parameter estimates were averaged across models with AICc differences (Δ*i *< 2) correcting for model weights (Burnham & Anderson, 2002). All statistical analyses were performed in R 3.4.1 (R Development Core Team, 2016). Descriptive statistics are presented as the mean ± standard deviation, whereas the parameter estimates are presented with their 95% confidence intervals (CI) and are considered to be statistically significant if the 95% CI did not overlap zero.

## 3 RESULTS

In total, 33 females with 55 litters were recorded but of these sufficient spatial data (>three sightings) were only available for 20 individuals (Table S3). The number of cubs that were recruited per litter ranged from 0 to 4, and on average, 1.71 ± 1.35 cubs were recruited per litter. The top model (AIC < 2) that best explained the variation in cub recruitment included the proportion of open habitat within a 1,440 m radius and tourist abundance (Table 1). Lion and spotted hyaena abundance were not present in the top models.

**Table 1:** Summary of model selection statistics for general linear mixed models used to determine cheetah cub recruitment in relation to the proportion of open habitat within a 1,440 m radius and the abundance of tourists, lions, and spotted hyaenas

Model	LL	AICc	Δ_*i*_	w_*i*_
Open_1,440 m_ + Tourist	−492.965	996.0	0.00	0.838
Tourist	−495.856	999.8	3.74	0.129
Open_1,440 m_ + Tourist + Lion	−495.903	1004.0	7.92	0.016
Open_1,440 m_ + Tourist + Hyaena	−496.277	1004.7	8.67	0.011
Tourist + Lion	−498.527	1007.2	11.12	0.003
Tourist + Hyaena	−499.111	1008.3	12.29	0.002
Open_1,440 m_	−501.195	1010.5	14.42	0.001
Open_1,440 m_ + Tourist + Lion + Hyaena	−499.134	1012.5	16.44	0.000
Tourist + Lion + Hyaena	−501.658	1015.5	19.43	0.000
Open_1,440 m_ + Lion	−503.962	1018.0	21.99	0.000
Open_1,440 m_ + Hyaena	−504.497	1019.1	23.06	0.000
Lion	−508.485	1025.1	29.00	0.000
Hyaena	−508.740	1025.6	29.51	0.000
Open_1,440 m_ + Lion + Hyaena	−507.326	1026.8	30.77	0.000
Lion + Hyaena	−511.762	1033.6	37.59	0.000

Models were ranked according to the Akaike information criterion corrected for small sample size (AICc).

Included are the log‐likelihood (LL), the AICc values, the AICc differences (Δ*i*), and the Akaike weights (*wi*).

**Figure 2:**
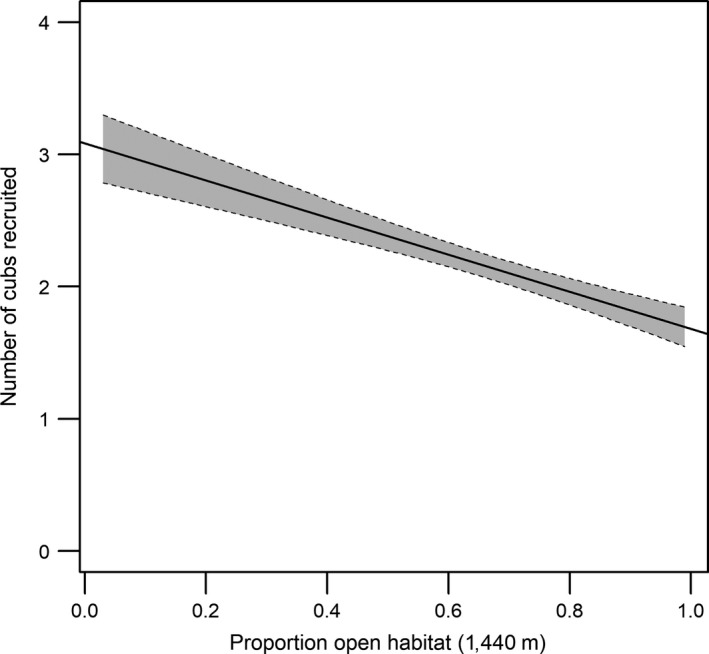
The effect of the proportion of open habitat present within a 1,440 m radius on the number of cheetah cubs that are recruited per litter in the Maasai Mara, Kenya. The shaded area represents the 95% confidence interval

**Figure 3:**
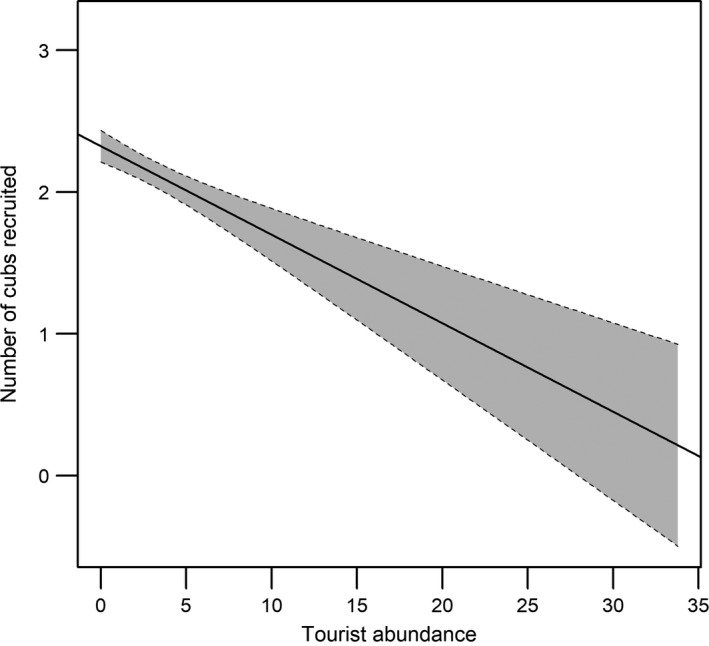
The effect of the abundance of tourist vehicles on the number of cheetah cubs that are recruited per litter in the Maasai Mara, Kenya. The shaded area represents the 95% confidence interval

The amount of open habitat that was present within a 1,440 m radius had a negative effect on average cub recruitment (estimate = −0.415, CI = −0.706 to −0.123, Figure 2). In other words, female cheetahs who were mostly found in areas where there was a high proportion of open habitat on average raised 1.69 ± 0.14 cubs per litter to independence whereas females who were mostly found in less open habitats on average recruited 3.04 ± 0.26 cubs per litter. Similarly, tourist abundance had a negative effect on cheetah cub recruitment (estimate = −0.037, CI = −0.052 to −0.022, Figure 3). Female cheetahs that were exposed to high levels of tourism on average raised 0.21 ± 0.72 cubs per litter to independence compared to 2.32 ± 0.11 cubs for females that were generally exposed to low levels of tourism.

## 4 DISCUSSION

My results show that the number of cubs that were raised to independence was negatively affected by the amount of open habitat and the abundance of tourists. These results have important implications for regulating the volume of tourism and indicate that habitat heterogeneity is desirable in the existing cheetah range and should be considered in habitat restoration and cheetah reintroduction programs.

Open habitats can be risky as the possibilities of being able to hide from predators are limited, whereas areas with vegetative cover can act as a “refuge” by providing concealment (Janssen, Sabelis, Magalhães, Montserrat, & Hammen, 2007). For species that experience predator‐mediated mortality, such as cheetahs, it is likely that the availability and accessibility of competition refuges is an important factor determining offspring survival and recruitment. For example, the survival of swift foxes (*Vulpus velox*) in Canada was lower than in Mexico because fewer refuges were available to avoid coyotes (*C. latrans*; Moehrenschlager, List, & Macdonald, 2007). Similarly, Nafus, Esque, Averill‐Murray, Nussear, and Swaisgood (2017) found that the availability of burrows, which aided concealment, improved the juvenile survival of Mojave desert tortoises (*Gopherus agassizii*).

In addition to predation, cheetahs often fall victim to kleptoparasitism, where their kills are stolen by other predators. In the Maasai Mara, 12% of cheetah kills are stolen (Broekhuis, Thuo, & Hayward, 2018) which could have an indirect effect on survival if the necessary energy requirements are not reached (Frid & Dill, 2002). This has been observed in other carnivores, such as leopards *(P. pardus)*, where females that suffered from a high rate of kleptoparasitism had lower reproductive success (Balme, Miller, Pitman, & Hunter, 2017). However, the chance of falling victim to kleptoparasitism is reduced in areas with dense vegetation (Hunter, Durant, & Caro, 2007a; Paulson, 1985) and it is possible that areas with open habitat experience reduced cub recruitment due to predation and kleptoparastism. That cheetah cub recruitment increases in denser habitat might be counterintuitive as predators, such as lions, prefer dense vegetation (Broekhuis et al., 2013). However, because cub recruitment is higher in habitats that are preferred by lions, it strengthens the support that dense habitat is important for concealment and hence recruitment.

While dense habitat plays an important role in cub recruitment, cheetahs also use open grasslands (Broekhuis et al., 2013). As different habitats fulfill different ecological requirements, maintaining habitat heterogeneity could be important to cheetah survival. Prescribed burning is common practice throughout Africa as a tool to improve forage and enhance wildlife viewing (Green, Roloff, Heath, & Holekamp, 2015). In the Maasai Mara, burning has been shown to increase both herbivore and carnivore abundance in areas postburn. However, burning can accelerate habitat conversion and increase habitat homogeneity, while attracting small ungulates (Anderson et al., 2016), the preferred prey species of female cheetahs with cubs (Broekhuis et al., 2018). Burning could therefore create an “ecological trap” by attracting female cheetahs to open areas where prey abundance is high, but cub recruitment is low (Schlaepfer, Runge, & Sherman, 2002).

Spatially heterogeneous environments are key in source–sink dynamics (Pulliam & Danielson, 1991), which could be important for the persistence of cheetah populations. For example, it has been suggested that cheetah cub mortality in the Serengeti National Park could be uncharacteristically high due to lack of vegetative cover and Laurenson (1995) found that cheetah recruitment on the Serengeti Plains was not enough to maintain the population. My results indicate that open areas could be a sink and that female cheetahs residing in denser habitat types are likely to act as a source. Therefore, it is possible that in the Serengeti‐Mara ecosystem, the viability of the cheetah population depends on cub recruitment in other, more densely vegetated, areas. This source–sink dynamic could also explain why, in the Serengeti, overall lion numbers do not have a negative effect on the cheetah population as whole (Swanson et al., 2014), despite the fact that lion abundance is negatively correlated with the survival and recruitment of cheetah cubs (Durant et al., 2004). In the present study, lion nor spotted hyaena abundance had a negative effect on cheetah cub recruitment despite other studies showing that cheetah cubs are often killed by these predators (Laurenson, 1994) and that a larger number of cubs per litter are recruited in areas without lions compared to areas with lions (Bissett & Bernard, 2011). It is therefore possible that the mere presence of other predators, rather than actual abundance, has a negative impact on cheetah cub recruitment. This, however, needs further investigation.

In natural systems, lions are considered to be the top predators but, in an increasingly anthropogenic world, humans could take this title as smaller predators may react more strongly to humans than to natural predators (Clinchy et al., 2016). In this study, a negative relationship was observed between the average number of cubs recruited and the abundance of tourist vehicles. In Russia, tiger cub survival was similarly found to be negatively affected by increased human presence in protected areas which was partly because of mortalities caused by vehicle collisions (Kerley et al., 2002). Although cubs have been reported to be killed by tourists vehicles in the Serengeti (Durant et al., 2010), there is currently no hard evidence of this happening in the Maasai Mara. It is therefore likely that tourists are having an indirect effect. Cheetahs, especially with cubs, are a major tourist attraction and commonly attract large numbers of vehicles (during this study, we observed a case of 64 vehicles present at one sighting over a period of 2 hr). High tourist numbers have been found to negatively impact cheetah hunts (Burney, 1980), and even if a hunt is successful, the presence of tourists can result in a cheetah abandoning its kill (Broekhuis et al., 2018; Hunter, Durant, & Caro, 2007b). If a female cheetah with cubs cannot forage effectively, then this could have an indirect impact on her offspring (Frid & Dill, 2002; Laurenson, 1994)**.** Human disturbance can also cause changes in behavior (Elowe & Dodge, 1989) and increase stress levels (Creel et al., 2002; Hayward & Hayward, 2009) which in turn could influence fitness (Tuomainen & Candolin, 2011). For example, juvenile hoatzins (*Opisthocomus hoazin*) were found to be susceptible to tourist‐induced stress which was negatively correlated to their survival (Müllner, Linsenmair, & Wikelski, 2004). The availability of refuges, such as dense vegetation, could therefore not only be important in minimizing detections by predators, but also humans. Such is the case for wolves who were found to strongly select for refuges in a human‐dominated environment, allowing for co‐habitation (Llaneza, García, Palacios, Sazatornil, & López‐Bao, 2016).

While this study shows that high tourist abundance has a negative impact on cheetah cub recruitment, it is important to note that tourism plays an important, positive role in cheetah conservation through, for example, the creation and maintenance of protected areas and wildlife conservancies (Buckley, Morrison, & Castley, 2016; Tablado & D’Amico, 2017) and positively influencing attitudes and behavioral intentions of local people toward predators (Broekhuis, Kaelo, Sakat, & Elliot, in press). However, the results presented here are worrying as growth rates for cheetahs inside the protected areas need to be high if they are to compensate for declines outside the protected areas (Durant et al., 2017). Therefore, if growth rates within protected areas are low due to decreased cub recruitment, the risk of extinction will increase. As cheetahs are a key species on tourists’ wish lists (Maciejewski & Kerley, 2014), a sustainable level of tourism needs to be found to ensure cheetah survival. Actions that could be taken to ensure that tourists do not have a negative impact on cheetahs include 1) allowing no more than five vehicles at a cheetah sighting, 2) ensuring that no tourist vehicles are allowed near a cheetah lair, 3) ensuring that vehicles keep a minimum distance of 30 m at a cheetah sighting, 4) ensuring that noise levels and general disturbance at sightings are kept to a minimum, 5) ensuring that vehicles do not separate mothers and cubs, and 6) ensuring that cheetahs on a kill are not enclosed by vehicles so that they can detect approaching danger. These guidelines could be incorporated into management policies and distributed to tourists upon arrival. Rangers could then ensure the policies are upheld.

In summary, the results in this study illustrate the negative effect of open habitat and tourist abundance on cheetah cub recruitment. The findings that habitat and tourism have population level consequences are significant for cheetah conservation especially as numbers are continuing to decrease (Durant et al., 2017). The importance of a heterogeneous environment should therefore be taken into consideration in habitat management, restoration, and reintroduction programs. In addition, tourist quotas should be put in place in high visitation areas and strict wildlife viewing guidelines, such as number of vehicles, tourist behavior, time spent, and distance to a sighting, should be enforced. Cub recruitment is an important component of species persistence and understanding natural and human influences on such vital rates could aid conservation efforts.

## CONFLICT OF INTEREST

None declared.

## AUTHOR CONTRIBUTION

FB conceived the idea, analyzed the data, and wrote the manuscript.
