# Supplementary material for: Natural and anthropogenic drivers of cub recruitment in a large carnivore
Source: Ecol Evol. 2018 Jun 17;8(13):6748–55. doi: 10.1002/ece3.4180 (PMC6053572; doi:10.1002/ece3.4180)
Supplement: Supplementary file 1 [file ECE3-8-6748-s001.docx]

| **Habitat structure** | **Habitat type** | **Description** | **Percentage of study area** |
| --- | --- | --- | --- |
| **Open** | Bare ground | Areas with no vegetation i.e. rocky outcrops, eroded areas, roads etc. | 66.23% |
|  | Grassland | Open grasslands with the occasional tree or clump of bushes. Grass species incl. Red oat grass (*Themeda triandra*), Thatch grass (*Hyparrhenia rufa*) and Sweet pitted grass (*Bothriochloa insculpa*) and tree species incl. Vachellia sp., Balanites |  |
| **Semi-closed** | Mixed scrub | Vachellia and different bushes | 32.70% |
|  | Bushes | Orange-leaved croton (*Croton dichogamus*), Ol Kinyei (*Eulclea divinorum*) |  |
|  | Whistling thorn | Whistling thorn (*Vachellia drepanolobium*) |  |
|  | Vachellia woodland | Incl. various Vachellia sp. Often open understory with tall trees |  |
|  | Riparian | Dense woodland vegetation. Species incl. *Warburgia ugandensis, Vachellia xanthophloea, Euclea divinorum* and *Tarchonanthus camphoratus.* |  |
| **Other** | No data | Clouds and water | 1.07 % |

**Table S1.** Summary of the different habitat types found in the Maasai Mara, Kenya. Habitat classification was based on two different habitat structures; open and semi-closed*.

* The habitat map was created based on two LandSat 8 images, one from 17^th^ July 2013 and one from 25^th^ January 2014. Both had a 30m spatial resolution. The images were classified based on habitat structure using the Random Forest method, chosen for its high classification accuracy ([Cutler et al. 2007](#_ENREF_1); [Kampichler et al. 2010](#_ENREF_2)). The training data was created in Quantum GIS v2.8.4 ([QGIS Development Team 2015](#_ENREF_3)) using a combination of 378 habitat points obtained on the ground and high resolution SPOT 5 imagery (2.5m resolution) from 2011. In addition to the original satellite images, we also used the Normalised Difference Vegetation Index (NDVI) and texture to increase the accuracy of classification. The classification was carried out using the ‘*randomForest*’ package in the statistical software R 2.14.2 ([R Development Core Team 2016](#_ENREF_4)). The final map was ground-truthed based on 2000 points created in Quantum GIS v2.8.4 ([QGIS Development Team 2015](#_ENREF_3)) and the final habitat map had an accuracy of 87%.

**References**

Cutler, D.R., Edwards Jr, T.C., Beard, K.H., Cutler, A., Hess, K.T., Gibson, J. & Lawler, J.J. (2007) Random forests for classification in ecology. *Ecology,* **88,** 2783-2792.

Kampichler, C., Wieland, R., Calmé, S., Weissenberger, H. & Arriaga-Weiss, S. (2010) Classification in conservation biology: A comparison of five machine-learning methods. *Ecological Informatics,* **5,** 441-450.

QGIS Development Team (2015) QGIS Geographic Information System. Open Source Geospatial Foundation Project [http://qgis.osgeo.org](http://qgis.osgeo.org/).

R Development Core Team (2016) R: A language and environment for statistical computing. R Foundation for Statistical Computing, Vienna, Austria.
