# Supplementary material for: Natural and anthropogenic drivers of cub recruitment in a large carnivore
Source: Ecol Evol. 2018 Jun 17;8(13):6748–55. doi: 10.1002/ece3.4180 (PMC6053572; doi:10.1002/ece3.4180)
Supplement: Supplementary file 2 [file ECE3-8-6748-s002.docx]

**Table S2**. Summary of the univariate scaling analysis which was performed for open and semi-closed habitat to determine the scale that had the strongest relationship with cheetah cub recruitment in the Maasai Mara, Kenya. Model selection was used to identify the most supported scale for each variable based on Akaike Information Criterion corrected for small sample size (AICc). For each habitat category we retained only the scale with the lowest AICc score for the multivariate analysis. Included are the log likelihood (LL), the AICc values, the AICc differences (Δ*i*) and the Akaike weights (*wi*).

| **Habitat category** | **Scale (m)** | **LL** | **AICc** | **Δ*i*** | **w*i*** |
| --- | --- | --- | --- | --- | --- |
| Open | 1440 | -501.19 | 1010.5 | 0.00 | 0.360 |
| Semi-closed | 1440 | -501.59 | 1011.3 | 0.79 | 0.243 |
| Open | 720 | -502.19 | 1012.5 | 1.99 | 0.133 |
| Semi-closed | 720 | -502.20 | 1012.5 | 2.00 | 0.132 |
| Semi-closed | 2880 | -503.22 | 1014.5 | 4.06 | 0.047 |
| Open | 2880 | -503.97 | 1016.0 | 5.55 | 0.022 |
| Semi-closed | 180 | -504.35 | 1016.8 | 6.31 | 0.015 |
| Open | 180 | -504.46 | 1017.0 | 6.53 | 0.014 |
| Semi-closed | 360 | -504.48 | 1017.0 | 6.57 | 0.014 |
| Open | 360 | -504.53 | 1017.1 | 6.67 | 0.013 |
| Semi-closed | 90 | -505.81 | 1019.7 | 9.22 | 0.004 |
| Open | 90 | -505.81 | 1019.7 | 9.23 | 0.004 |
