# Supplementary material for: Natural and anthropogenic drivers of cub recruitment in a large carnivore
Source: Ecol Evol. 2018 Jun 17;8(13):6748–55. doi: 10.1002/ece3.4180 (PMC6053572; doi:10.1002/ece3.4180)
Supplement: Supplementary file 3 [file ECE3-8-6748-s003.docx]

| **Cheetah ID** | **Number of litters** | **Total number of cubs recruited** | **Number of data points** |
| --- | --- | --- | --- |
| F001 | 4 | 6 | 60 |
| F002 | 1 | 2 | 35 |
| F004 | 2 | 3 | 127 |
| F005 | 1 | 1 | 28 |
| F006 | 2 | 3 | 29 |
| F007 | 1 | 3 | 3 |
| F008 | 2 | 1 | 9 |
| F010 | 2 | 5 | 33 |
| F016 | 2 | 3 | 56 |
| F020 | 1 | 1 | 4 |
| F021 | 2 | 4 | 4 |
| F024 | 1 | 4 | 3 |
| F026 | 3 | 3 | 39 |
| F027 | 1 | 4 | 51 |
| F028 | 1 | 3 | 5 |
| F029 | 1 | 0 | 9 |
| F039 | 1 | 3 | 3 |
| F042 | 1 | 0 | 4 |
| F044 | 1 | 0 | 3 |
| F045 | 1 | 4 | 3 |

**Table S3**. Summary of the data that were used to determine the factors affecting cheetah cub recruitment in the Maasai Mara, Kenya.
